# Amelioration of Renal Injury by Resveratrol in a Rat Renal Transplantation Model via Activation of the SIRT1/NF-*κ*B Signaling Pathway

**DOI:** 10.1155/2022/7140961

**Published:** 2022-03-28

**Authors:** Qin Zhou, Jiamin Li, Zhicong Xiang, Hequn Zou, Xiaofei Shao

**Affiliations:** ^1^Department of Nephrology, The Third Affiliated Hospital, Southern Medical University, Guangzhou 510660, China; ^2^Department of Nephrology, Pinghu Hospital, Health Science Center, South China Hospital of Shenzhen University, Shenzhen 518116, China

## Abstract

**Purpose:**

The improvement of the long-term survival of patients receiving kidney transplantation remains challenging. Ischemia reperfusion injury (IRI) reduces long-term renal graft survival in the early posttransplantation phase. However, few studies have investigated the effects of IRI on the pathogenesis of chronic renal graft failure. Silent information regulator 1 (SIRT1) regulates antioxidative stress and inflammatory response and protects against IRI. This study is aimed at investigating the role of resveratrol (RSV), an SIRT1 activator, in preventing renal injury in a rat renal transplantation model.

**Methods:**

A classical F334-to-LEW orthotopic renal transplantation rat model was established. The experiment group was treated with RSV from three days prior to kidney transplantation and the treatment lasted until the day of harvest. Uninephrectomized F344 and Lewis rats were used as controls. After 12 weeks, the effects of RSV were evaluated according to renal function, histopathology, immunohistochemistry, and western blotting. The activities of oxidative stress-related markers and proinflammatory cytokines were also assessed.

**Results:**

RSV treatment significantly ameliorated renal function and histopathological lesions in kidney-transplanted rats and increased the levels of GSH, SOD, and CAT and decreased the levels of MDA and iNOS. Furthermore, RSV also inhibited the expression of proinflammatory cytokines/chemokines such as TNF-*α*, CD68, and IL-6 in kidney-transplanted rats. In addition, the transplant group displayed significantly lower level of SIRT1 and higher level of Ac-NF-*κ*Bp65. RSV increased the expression of SIRT1 and decreased the expression of Ac-NF-*κ*Bp65.

**Conclusion:**

SIRT1 plays an important role in the pathogenesis of chronic renal allograft dysfunction. It is a potential therapeutic agent for ameliorating inflammation and oxidative stress-induced renal injury following kidney transplantation by activating the SIRT1/NF-*κ*B signaling pathway.

## 1. Introduction

Kidney transplantation is the most effective treatment for patients with end-stage renal disease. In recent decades, the application of immunosuppressive agents has reduced the occurrence of acute rejection of transplanted kidneys [1]. However, ensuring the long-term survival of a transplanted kidney remains challenging. Ischemia reperfusion injury (IRI) reduces long-term renal graft survival. Ischemia is considered an inevitable event following kidney transplantation. Early IRI can contribute to late graft loss by reducing renal functional mass and causing graft vascular injuries and chronic hypoxia, as well as subsequent fibrosis [2]. IRI involves various pathophysiological processes, such as oxidative stress injury, intracellular inflammatory response, and cell apoptosis [3]. Accordingly, it is necessary to take effective measures to attenuate inflammation reactions and oxidative stress injuries caused by IRI following kidney transplantation.

Silent information regulator 1 (SIRT1), a member of the sirtuin protein family, is a nicotinamide adenine dinucleotide+- (NAD+-) dependent deacetylase. This protein regulates both histone acetylation and the activities of several transcriptional factors, such as p53, nuclear factor-NF-*κ*B (NF-*κ*B), and forkhead box type O transcription factors (FOXOs) [4]. NF-*κ*B plays a central role in inflammation, and acetylation of the NF-*κ*B subunit RelA/p65 at lysine310 is related to the activity of NF-*κ*B and other inflammatory transcription factors [5, 6]. SIRT1 negatively regulates inflammation and exerts anti-inflammatory effects. The activation of SIRT1 can inhibit the production of tumor necrosis factor- (TNF-) *α*, monocyte chemoattractant protein 1, and interleukin 6 (IL-6) via inhibiting NF-*κ*Bp65 acetylation [7]. Furthermore, SIRT1 is the most widely studied sirtuin protein in the kidney. It exerts cytoprotective effects by inhibiting cell apoptosis, inflammation, and fibrosis in various kidney diseases such as diabetic nephropathy, acute kidney injury, and renal fibrosis [8–10]. Several recent studies showed that the activation of SIRT1 can protect against IRI in the liver, heart, brain, and kidney [11–14]. It was also suggested that SIRT1 is involved in the pathogenic process of interstitial fibrosis and inflammation during the early stage of chronic renal allograft dysfunction [15]. However, the underlying mechanism of SIRT1 remains unclear.

Resveratrol (RSV) (3,5,4′-trihydroxy-trans-stilbene), a natural polyphenolic compound found in grapes, berries, and many other plant species [16], is well known for its antioxidant properties. It is also known as an activator of the SIRT1 signaling pathway. Resveratrol ameliorates sevoflurane-induced cognitive impairment by activating the SIRT1/NF-*κ*B pathway in neonatal mice [17]. We hypothesized that RSV pretreatment could ameliorate renal injuries caused by kidney transplantation by activating the SIRT1/NF-*κ*B signaling pathway.

## 2. Material and Methods

### 2.1. Animals

Fisher (F344) and Lewis (LEW) rats, weighing 150–220 g and aged 8–12 weeks, were purchased from the Charles River Laboratories, Inc. (Beijing, China). The rats were housed in a specific pathogen-free laboratory with controlled temperature and humidity under 12 h light/dark cycles at the Animal Experimental Center of Southern Medical University (Guangzhou, China). The study was approved by the Committee of the Ethics of Animal Experiments of the Southern Medical University (permission no. 00119486), and all experiments were performed in accordance with the Guide for the Care and Use of Laboratory Animals published by the United States National Institutes of Health [18].

### 2.2. Experimental Design

The experimental rats were divided into four groups: (1) F344 control group, consisting of uninephrectomized F344 rats (*n* = 6); (2) LEW control group, consisting of uninephrectomized LEW rats (*n* = 6); (3) transplant group, consisting of LEW rats that underwent orthotopic left kidney transplantation from F334 donors (*n* = 6); and (4) RSV group, consisting of transplant recipient LEW rats treated with RSV (30 mg·kg^−1^·d^−1^; Sigma, St. Louis, Missouri, USA) via intraperitoneal injection (*n* = 6). The treatment was started three days prior to kidney transplantation and lasted until the day of harvest. The kidneys of rats in all groups were harvested at week 12 after renal transplantation. As RSV was dissolved in 4% dimethyl sulfoxide (Sigma, St. Louis, Missouri, USA), rats in the other three groups were also administered with 4% dimethyl sulfoxide to eliminate its effects.

### 2.3. Kidney Transplantation

F334-to-LEW orthotopic renal transplantation was performed as previously described under a surgical ASX-2 microscope (Shanghai Anxin Optical Instrument Manufacture Co., Ltd., Shanghai, China) [19]. Rats were anesthetized with 3% sodium pentobarbital (30 mg·kg^−1^) via intraperitoneal injection. For each recipient LEW rat, the left renal vessels were clamped, and the left kidney was isolated. The left donor kidney from an F344 rat was then isolated, cooled, and positioned orthotopically in the recipient LEW rat. The renal arteries, veins, and ureters of the donor and recipient were then linked end-to-end with 10-0 prolene (Ningbo Lingqiao Biological Technology, Ningbo, China). A low dose of cyclosporine A (1.5 mg·kg^−1^·d^−1^; Novartis International AG, Basel, Switzerland) was administered for 10 days following transplantation to suppress acute rejection. To prevent infection, recipients were intraperitoneally injected with ceftriaxone (20 mg·kg^−1^·d^−1^) for three days. The right native kidney of each recipient was removed on day 10 after renal transplantation. Rats showing overt signs of unsuccessful surgeries were excluded from the study. The model of rat kidney transplantation was associated with high survival rate (85.7%); out of a total of 14 kidney-transplanted rats, 2 had showed overt signs of unsuccessful surgery and 12 had been successfully used for further experimentation.

### 2.4. Uninephrectomy Surgery

Rats in the F344 and LEW control groups were anesthetized as described above. In each case, the right kidney was pulled out of the abdomen by holding the perirenal fat at the lower pole with blunt forceps. Next, ligation was conducted for the renal artery, vein, and ureter (~0.5 cm below the level of the hilum) with nonabsorbable surgical suture threads, after which the kidney was snap resected. The uninephrectomized rats were given ceftriaxone via intraperitoneal injection for three days. The survival rates for uninephrectomized rats were 100%, and there were 6 rats in each control group.

### 2.5. Tissue Harvest and Assessment of Kidney Function

The rats in all groups were anesthetized with sodium pentobarbital (intraperitoneal injection of 60 mg·kg^−1^) and subjected to cardiac exsanguination at week 12 after surgery. Blood and kidney samples of all rats were collected for further tests. Serum samples of the rats were used to evaluate the blood urea nitrogen and serum creatinine levels using an automatic biochemical analyzer (AU5400; Olympus, Tokyo, Japan). The kidney of each rat was cut into two sections. One section was stored at -80°C in liquid nitrogen for western blot analysis, whereas the other was fixed in 4% formalin for histopathological and immunohistochemical analyses.

### 2.6. Renal Histopathology

Kidney tissues were fixed in 4% paraformaldehyde at room temperature for 24 h and then embedded in paraffin. Paraffin sections (2 *μ*m) were stained with hematoxylin and eosin (HE) and with Masson's trichrome, which were then observed under a light microscope. The score of each kidney section was calculated as the average of ten random high-power fields. Renal damage was semiquantitatively scored on a scale of 0–3 for interstitial cellular infiltration, tubulopathy/interstitial fibrosis, glomerulopathy, and arterial intimal fibroplasia based on the Banff 2015 criteria [20]. The total Banff score (0-12) was calculated for each sample.

### 2.7. Immunohistochemistry

Kidney tissues embedded in paraffin were cut into 4 *μ*m slices. Antigens were retrieved using the citric acid buffer (pH 6.0) microwave antigen retrieval method and then detected via PV9000 two-stage staining (PV-9000 kit, Zhongshan Golden Bridge Biotechnology Company Co., Ltd., Beijing, China). H_2_0_2_ (3%) was used to block endogenous peroxidase. The sections were then incubated with primary antibodies including mouse monoclonal anti-SIRT1 antibody (1 : 100, ab110304, Abcam, Cambridge, UK), rabbit polyclonal anti-NF-*κ*Bp65 antibody (1 : 100, ab16502, Abcam), mouse monoclonal anti-TNF-*α* antibody (1 : 100, s-52746, Santa Cruz Biotechnology, Dallas, TX, USA), and rabbit polyclonal anti-CD68 antibody (1 : 100, ab125212, Abcam) at 4°C overnight. After bewashing with phosphate-buffered saline three times, the sections were incubated with a secondary antibody for 20 min at room temperature. Finally, the sections were stained with diaminobenzidine color reagent (Golden Bridge Biotechnology Co., Ltd., Beijing, China). The intensity of positive staining was semiquantified using Image-Pro Plus 7.0 software (Media Cybernetics, Rockville, MD, USA).

### 2.8. Assessment of Oxidative Stress

A series of commercial kits (Nanjing Jiancheng Bioengineering Institute, Nanjing, China) were used to examine the activities or concentrations of superoxide dismutase (SOD), malondialdehyde (MDA), glutathione (GSH), and catalase (CAT) according to the manufacturer's instructions. Briefly, after the renal tissues were lysed, the protein concentration in each sample was calculated according to the standard curve, after which the samples were diluted with normal saline to a uniform concentration of 1 g·L^−1^. Next, each indicator was assessed according to the manufacturer's instructions.

### 2.9. Western Blot Analysis

Total protein was extracted from each kidney tissue sample using a commercial extraction kit (KeyGEN Biotech, Nanjing, China). The protein concentration was determined using a bicinchoninic acid protein assay kit (Beyotime Institute of Biotechnology, Shanghai, China). Protein samples (50 *μ*g·lane-1) were loaded onto a 10% sodium dodecyl sulfate-polyacrylamide gel. After the separation via electrophoresis, the samples were transferred onto polyvinylidene fluoride membranes (EMD Millipore, Billerica, MA, USA), which were then blocked with 5% bovine serum albumin (Sigma-Aldrich) in Tris-buffered saline containing Tween 20 at 4°C for 1 h. The membranes were washed three times with Tris-buffered saline containing Tween and incubated at 4°C overnight with primary antibodies against SIRT1 (1 : 2,000, Cell Signaling Technology), acetyl-NF-*κ*Bp65 (Lys310; 1 : 500, Cell Signaling Technology), NF-*κ*Bp65 (1 : 1000, Cell Signaling Technology), inducible nitric oxide synthase (iNOS; 1 : 1000, Santa Cruz Biotechnologies), superoxide dismutase 2 (SOD2; 1 : 1000, Abcam), TNF-*α* (1 : 1000, Abclonal, Wuhan, China), IL-6 (1 : 1000, Santa Cruz Biotechnologies), and glyceraldehyde-3-phosphate dehydrogenase (GAPDH; 1 : 5000, CW0100M, CWBIO, Beijing, China). The membranes were washed and then incubated with a horseradish peroxidase-conjugated secondary antibody (goat anti-mouse antibody or goat anti-rabbit antibodies, 1 : 5000, CW0102 or CW01035, respectively, CWBIO, Beijing, China) at room temperature for 1 h. The bands were observed using SuperSignal West Pico enhanced chemiluminescent substrate (Pierce, Rockford, IL, USA). Protein band intensities were quantified using ImageJ software version 1.46 (National Institute of Health, Bethesda, MD, USA).

### 2.10. Statistical Analysis

All data were expressed as the mean ± standard deviation (SD) and analyzed using Statistical Package for Social Sciences (SPSS) version 20 (SPSS, IBM, US). Kolmogorov-Smirnov tests were performed to assess the normality of the experimental data. One-way analysis of variance was performed to test statistical differences among treatments when the data were normally distributed; otherwise, the Kruskal-Wallis test was performed. *P* < 0.05 was considered to indicate a statistically significant difference.

## 3. Results

### 3.1. RSV Improves Renal Function

**Figure 1:**
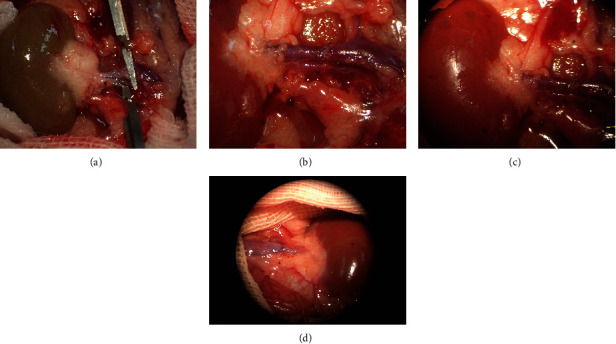
Orthotopic renal transplantation. (a) The transplanted kidney before reperfusion. (b) End-to-end anastomoses of renal vessels. (c) The transplanted kidney after reperfusion. (d) The survival of the transplant kidney after the right native kidney nephrectomy.

**Figure 2:**
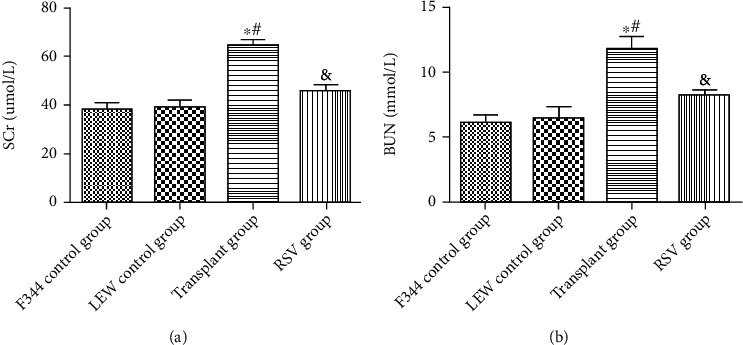
RSV improves renal function in kidney-transplanted rats. (a) Levels of serum creatinine. (b) Levels of blood urea nitrogen. Data are expressed as the mean ± SD. ^∗^*P* < 0.05 vs. the F344 control group, ^#^*P* < 0.05 vs. the LEW control group, and ^&^*P* < 0.05 vs. the transplant group.

Orthotopic kidney transplantation was successfully performed in rats (Figures 1(a)–1(d)). Serum creatinine and blood urea nitrogen are important indicators of renal function. As shown in Figure 2, compared to in the F344 and LEW control rats, the levels of serum creatinine and blood urea nitrogen were significantly higher in the transplant group than in the F344 and LEW control groups (*P* < 0.05). Furthermore, the levels of serum creatinine and blood urea nitrogen were lower in the RSV group than in the transplant group (*P* < 0.05).

### 3.2. RSV Ameliorates Renal Histological Damage in Kidney-Transplanted Rats

**Figure 3:**
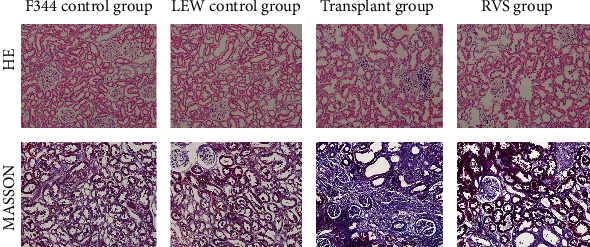
Photomicrographs illustrating HE staining and Masson's trichrome staining of kidney tissues from different groups (original magnification, 200x).

**Figure 4:**
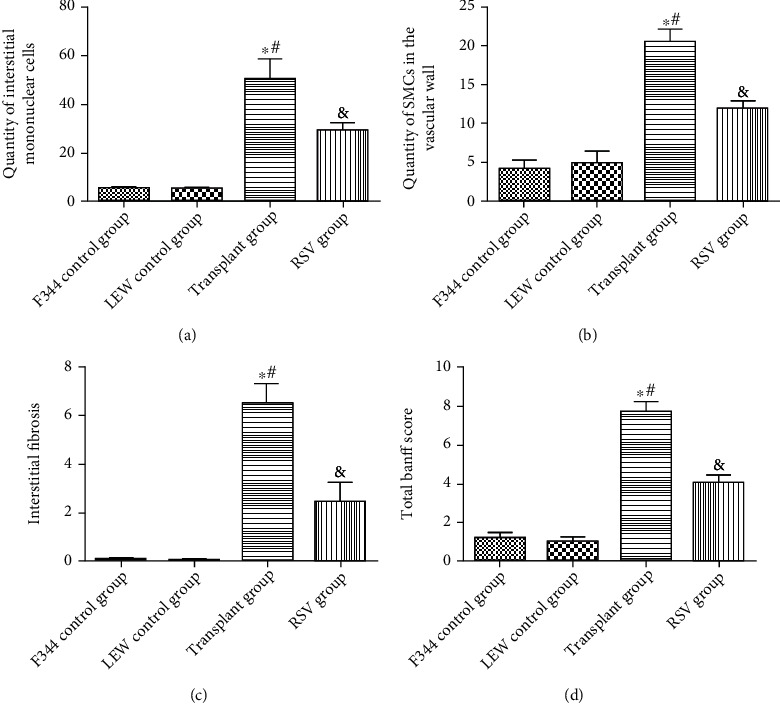
RSV ameliorates renal histological damage in transplant rats. (a) Quantity of interstitial mononuclear cells. (b) Quantity of smooth muscle cells in the vascular wall. (c) Interstitial fibrosis. (d) Total Banff score. Data are expressed as the mean ± SD. ^∗^*P* < 0.05 vs. the F344 control group, ^#^*P* < 0.05 vs. the LEW control group, and ^&^*P* < 0.05 vs. the transplant group.

The primary pathological changes in transplanted kidneys included mononuclear cell infiltration, smooth muscular cell migration, renal vessel sclerosis, and tubulopathy/tubulointerstitial fibrosis. Histological changes that occurred in the kidneys of the four groups are shown in Figure 3. The F344 and LEW control groups did not show significant morphological changes. In contrast, the kidneys in the transplant group showed interstitial mononuclear cell infiltration, intimal proliferation, smooth muscle cell migration, and interstitial fibrosis. As shown in Figure 4, the Banff scores of the transplant group were significantly higher than those of the other groups (*P* < 0.05). RSV treatment alleviated these lesions and tissue damage as well as reduced the total Banff scores (*P* < 0.05).

### 3.3. RSV Inhibits Oxidative Stress in the Renal Tissues of Kidney-Transplanted Rats

**Figure 5:**
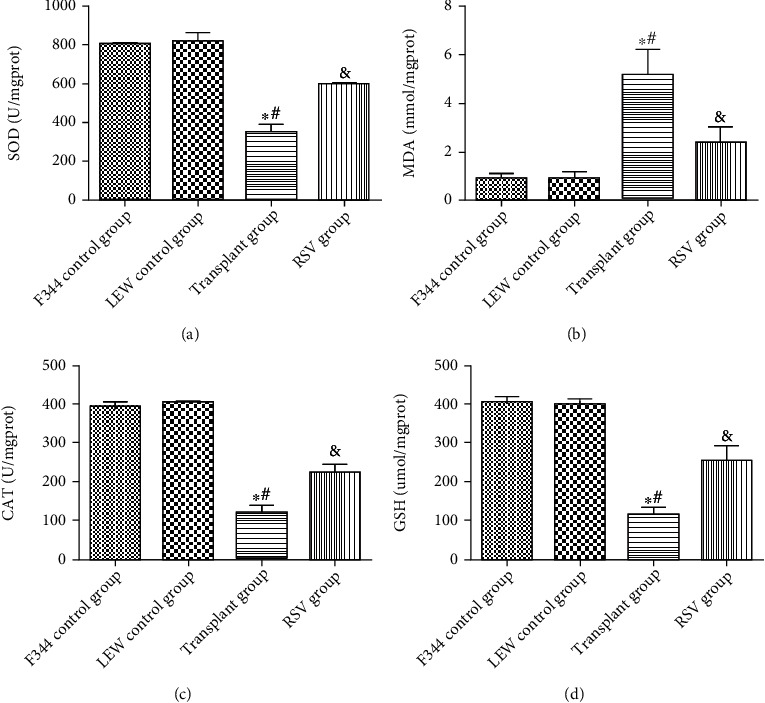
RSV inhibits oxidative stress in renal tissues of kidney-transplanted rats. (a) SOD activity. (b) MDA level. (c) CAT activity. (d) GSH level. Data are expressed as the mean ± SD. ^∗^*P* < 0.05 vs. the F344 control group, ^#^*P* < 0.05 vs. the LEW control group, and ^&^*P* < 0.05 vs. the transplant group.

**Figure 6:**
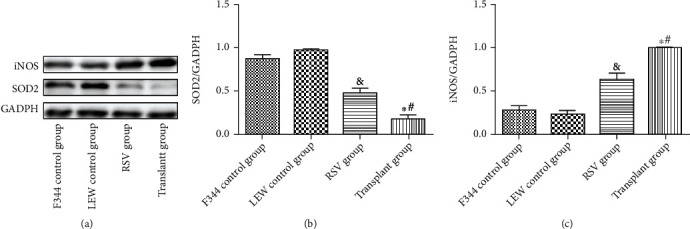
Protein levels in different groups. (a) Western blot of iNOS and SOD2. (b) Quantitative analysis of SOD2. (c) Quantitative analysis of iNOS. Data are expressed as the mean ± SD. ^∗^*P* < 0.05 vs. the F344 control group, ^#^*P* < 0.05 vs. the LEW control group, and ^&^*P* < 0.05 vs. the transplant group.

As shown in Figure 5, the transplant group showed higher levels of malondialdehyde (MDA) and lower levels of glutathione (GSH), superoxide dismutase (SOD), and catalase (CAT) activity than the F344 and LEW control groups (*P* < 0.05). RSV effectively reversed the enhancement of oxidative stress in kidney-transplanted rats (*P* < 0.05). As shown in Figure 6, compared with those in the F344 and LEW control groups, the levels of SOD_2_ were significantly lower in the transplant group, whereas the levels of inducible nitric oxide synthase (iNOS) in the transplant group were significantly higher than those in the F344 and LEW control groups. RSV treatment markedly abolished this effect (*P* < 0.05). These results demonstrate that RSV inhibited oxidative stress in renal tissues of kidney-transplanted rats.

### 3.4. RSV Attenuates Inflammatory Response in the Renal Tissues of Kidney-Transplanted Rats

**Figure 7:**
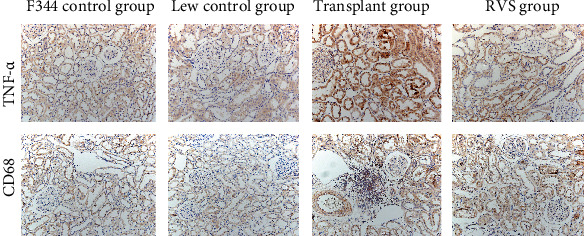
Photomicrographs illustrating immunohistochemistry staining of TNF-*α* and CD68 from kidney tissues of the different groups.

**Figure 8:**
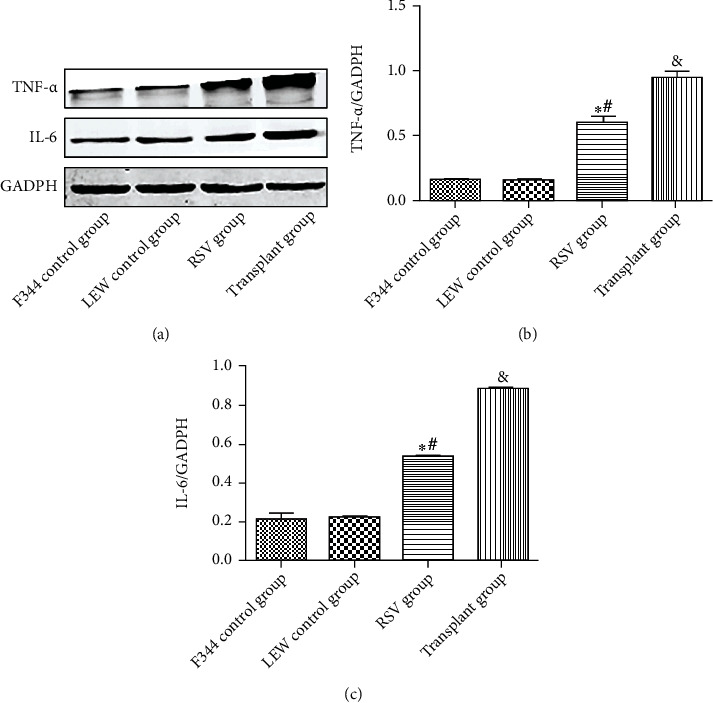
Protein levels in different groups. (a) Western blot of TNF-*α* and IL-6. (b) Quantitative analysis of TNF-*α*. (c) Quantitative analysis of IL-6. Data are expressed as the mean ± SD. ^∗^*P* < 0.05 vs. the F344 control group, ^#^*P* < 0.05 vs. the LEW control group, and ^&^*P* < 0.05 vs. the transplant group.

As shown in Figures 7 and 8, the levels of the inflammatory cytokines TNF-*α*, CD68, and IL-6 were significantly higher in the transplant group than those in the control groups (*P* < 0.05). Upregulation of TNF-*α*, CD68, and IL-6 was attenuated by RSV treatment (*P* < 0.05). These results reveal that RSV inhibits the inflammatory response in the renal tissues of kidney-transplanted rats.

### 3.5. RSV Elevates the Protein Levels of SIRT1 and Restrained the NF-*κ*B Signaling Pathway in the Renal Tissues of Kidney-Transplanted Rats

**Figure 9:**
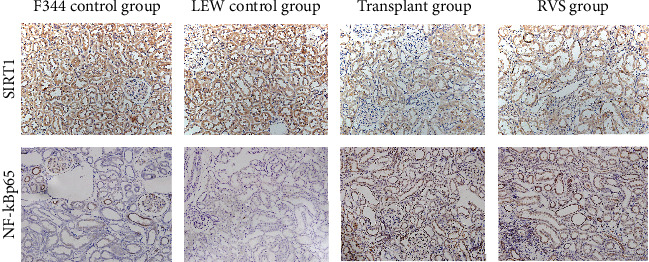
Photomicrographs illustrating immunohistochemistry staining of SIRT1 and NF-*κ*Bp65 from kidney tissues of different groups.

**Figure 10:**
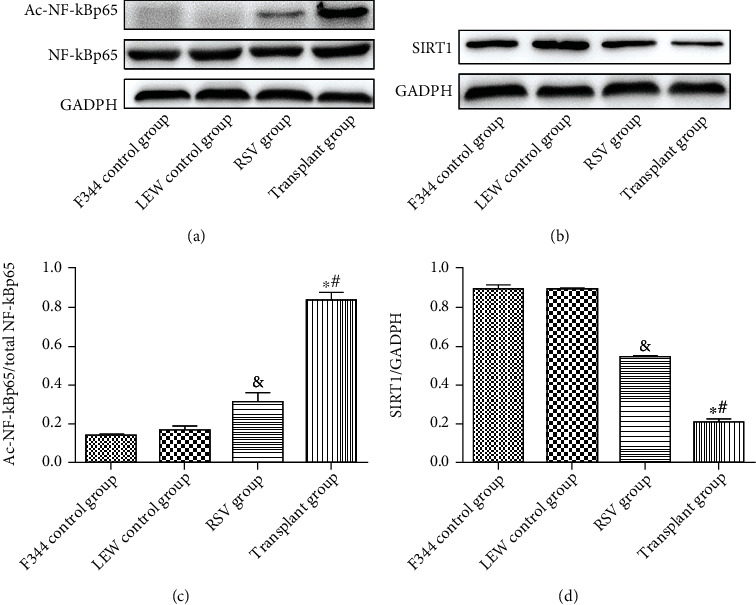
Protein levels in different groups. (a) Western blot of NF-*κ*Bp65 and Ac-NF-*κ*Bp65. (b) Western blot of SIRT1. (c) Quantitative analysis of Ac-NF-*κ*Bp65/total NF-*κ*Bp65. (d) Quantitative analysis of SIRT1. Data are expressed as the mean ± SD. ^∗^*P* < 0.05 vs. the F344 control group, ^#^*P* < 0.05 vs. the LEW control group, and ^&^*P* < 0.05 vs. the transplant group.

The transplant group displayed a significantly lower level of SIRT1 (*P* < 0.05, Figures 9 and 10) but a significantly higher level of Ac-NF-*κ*B (*P* < 0.05, Figures 10(a) and 10(c)) than the control groups. Furthermore, western blot analysis revealed that the Ac-NF-*κ*Bp65/total NF-*κ*Bp65 ratio was significantly higher in the transplant group than in the control groups (*P* < 0.05, Figures 10(a) and 10(c)). However, these effects were abolished by RSV treatment (*P* < 0.05). These findings demonstrate that RSV attenuates the suppression of Ac-NF-*κ*Bp65 expression by activating SIRT1.

## 4. Discussion

Renal transplantation has become an ideal treatment for patients with chronic renal failure. With the application of advanced immunosuppressive drugs, such as calcineurin inhibitors, acute rejection rates have decreased remarkably, and the one-year survival rate of renal grafts has increased to >90% [21]. However, most grafts develop chronic dysfunction, which adversely affects long-term graft survival. Additionally, renal grafts inevitably experience ischemia as soon as they are removed from the donor. IRI is a well-known risk factor for the development of delayed graft function [22, 23]. Although most renal graft transplant recipients can completely recover from the initial period, clinical evidence has demonstrated that transplanted kidneys with prolonged ischemic periods are more susceptible to long-term deterioration [24]. During kidney transplantation, the generation of reactive oxygen species and inflammatory cytokines caused by IRI relentlessly leads to monocyte infiltration, neutrophilia, renal endothelial injury, cell death, acute tubular necrosis, and microvascular injury [2]. Evidence has been shown that oxidative stress can activate a variety of transcription factors leading to the differential expression of some genes involved in inflammatory pathways such as TNF-*α*, IL-1*β*, and IL-6 [25, 26]. In our experiment, the kidneys in the transplant group showed interstitial mononuclear cell infiltration, intimal proliferation, smooth muscle cell migration, and interstitial fibrosis. Assessment of oxidative stress markers and inflammatory cytokines revealed that the levels of proinflammatory cytokines, such as TNF-*α*, CD68, and IL-6, were higher, whereas those of GSH, SOD, and CAT were lower in transplant rats than in control groups. These results demonstrate that oxidative stress and inflammation are involved in the pathogenesis of chronic renal allograft dysfunction, which is consistent with the findings of previous studies [15, 27, 28]. Pharmacological intervention plays a significant role in IRI damage. Unfortunately, however, effective clinical treatments for improving IRI damage during transplantation are currently unavailable.

SIRT1 is an NAD+-dependent deacetylase with numerous protective effects, including metabolic, oxidative, and hypoxic stress resistance and DNA repair [29]. Additionally, SIRT1 negatively regulates inflammation and exerts anti-inflammatory effects. A growing body of evidence suggests that SIRT1 plays an important role in preventing oxidative stress and inflammation [30–32]. Numerous studies have shown that SIRT1 is expressed in the kidney, and it protects the kidney from both chronic and acute kidney diseases, such as acute kidney injury, diabetic nephropathy, and renal fibrosis [33, 34]. The pharmacological activation of SIRT1 protects against ischemia/reperfusion-induced acute kidney injury [11, 35]. Additionally, it has been revealed that CYP2J2-produced epoxyeicosatrienoic acids protect against ischemia/reperfusion-induced kidney injuries by activating the SIRT1-FoxO3a signaling pathway [36]. The activation of SIRT1 plays a vital role in the treatment of renal ischemia/reperfusion injury. However, few studies have investigated the effects of SIRT1 on the pathogenesis of chronic renal graft failure. In a previous study, the potential role of SIRT1 in the pathogenic process of inflammation was investigated at the early stage of chronic renal allograft dysfunction [15]. However, the underlying action mechanism of SIRT1 is unclear. SIRT1 binds to and deacetylates various transcription factors, including nuclear factor E2-related factor 2, NF-*κ*B, pancreatic and duodenal homeobox factor 1, and FOXO. The transcription factor NF-*κ*B plays a critical role in regulating inflammation [37]. Deacetylation of p65 at Lys310 by SIRT1 controls NF-*κ*B transcriptional activity [38, 39]. The activation of SIRT1 also inhibits TNF-*α*-induced inflammation in fibroblasts by reducing acetylation of the NF-*κ*B subunit RelA/p65 [40]. In an in vivo study, collectively, the sevoflurane-induced inflammatory response was alleviated possibly via the modulation of the SIRT1/NF-*κ*B axis and microglial polarization in the hippocampus [41]. Acetylation at lysine 310 of NF-*κ*Bp65 has been reported to be associated with increased oxidative stress. SIRT1 activation by RSV leads to the deacetylation of both NF-*κ*Bp65 and H3, thereby attenuating cardiac oxidative stress and complications of diabetes [42]. SIRT1 inhibits the release of inflammatory factors via NF-*κ*Bp65 deacetylation in septic rats with acute kidney injury [43]. The present study shows that the expression level of SIRT1 was markedly lower, whereas that of Ac-NF-*κ*Bp65 was higher in the transplant group than in the control groups, which is consistent with the result in previous studies.

RSV is a phenolic compound that can increase the expression of SIRT1 and modulate the inflammation-oxidative stress cycle. The activation of SIRT-1 by RSV leads to deacetylation of both NF-*κ*Bp65 and H3, thereby attenuating cardiac oxidative stress and complications of diabetes [41]. RSV ameliorates sevoflurane-induced cognitive impairment by activating the SIRT1/NF-*κ*Bp65 pathway in neonatal mice [41]. Resveratrol induces chondrosarcoma cell apoptosis via a SIRT1-activated NF-*κ*B deacetylation and exhibits antichondrosarcoma activity in vivo [42]. We assessed whether RSV could reduce inflammation and oxidative stress injury by regulating the SIRT1/NF-*κ*Bp65 pathway in a rat kidney transplantation model. As expected, the transplant group displayed significantly lower levels of SIRT1 and higher levels of Ac-NF-*κ*Bp65 and multiple proinflammatory cytokines/chemokines; In addition, the levels of MDA and iNOS were higher, and those of GSH, SOD, and CAT were lower in the transplant group than in the control groups. The activation of SIRT1 via RSV pretreatment restored the expression of SIRT1 and decreased the expression of Ac-NF-*κ*Bp65. RSV inhibited oxidative stress and inflammatory response in renal tissues of kidney-transplanted rats. Furthermore, RSV improved renal function and alleviated renal histological damage in transplant rats. These findings suggest that RSV ameliorates renal inflammation and oxidative stress injury by activating the SIRT1/NF-*κ*B signaling pathway in a rat kidney transplantation model.

The limitation of our study is the dose of RSV pretreatment used. Further studies are required to evaluate the effects of different dosages of RSV. In addition, studies using SIRT1-knockout rat recipients should be performed to verify the direct or indirect effects of RSV.

## 5. Conclusion

Our results show that RSV pretreatment ameliorated renal inflammation and oxidative stress injury by activating the SIRT1/NF-*κ*B signaling pathway in a rat kidney transplantation model. Therefore, RSV may function as an ancillary therapeutic agent for preventing and treating chronic renal allograft dysfunction and which should be further evaluated.

## Data Availability

The data used to support the findings of this study are available from the corresponding author upon request.
